# Tirzepatide-induced weight loss and obstructive sleep apnea improvement in an adult with type 2 diabetes: A case report

**DOI:** 10.1097/MD.0000000000045445

**Published:** 2025-10-24

**Authors:** Noriko Funamizu, Naotake Funamizu, Tsunemichi Hirose

**Affiliations:** a Department of Gastroenterology, Hirose Hospital (Ehime), Imabari-City, Japan.

**Keywords:** case report, obesity, obstructive sleep apnea, tirzepatide, type 2 diabetes mellitus

## Abstract

**Rationale:**

Managing type 2 diabetes mellitus (T2DM) in adults with severe obesity frequently coexists with obstructive sleep apnea (OSA), complicating metabolic control and quality of life. Real-world, objective monitoring–documented trajectories through which pharmacologic weight loss enables withdrawal of continuous positive airway pressure (CPAP) remain clinically informative.

**Patient concerns:**

A 48-year-old man (height 178 cm; weight 129 kg; BMI 40.7 kg/m²) had T2DM and severe OSA diagnosed by polysomnography (PSG), with persistent daytime sleepiness despite CPAP and body-weight fluctuations between 108 and 129 kg.

**Diagnoses:**

T2DM with class III obesity and severe OSA with prior peak HbA1c of 8.2%.

**Interventions:**

Beginning in June 2023, tirzepatide was initiated with gradual dose escalation alongside nutritional counseling and light-to-moderate physical activity. At initiation, it was prescribed for glycemic control and weight reduction in T2DM and thus predated the subsequent U.S. FDA approval for OSA. OSA outcomes were later assessed with objective monitoring via CPAP telemonitoring as part of routine comorbidity care. CPAP was continued initially and reassessed after weight loss.

**Outcomes:**

Within 3 months, weight decreased to 108 kg (BMI 34.1 kg/m²) with improved glycemic control. On CPAP telemonitoring, the device-derived residual apnea–hypopnea index decreased and remained low over time. Given sustained weight loss and improved symptoms, CPAP was discontinued under supervision. At the most recent follow-up, the patient weighed 82 kg (BMI 25.9 kg/m²) and reported improved daytime functioning. No severe adverse events occurred.

**Lessons:**

In selected adults with T2DM and OSA, tirzepatide-associated weight loss may contribute to device-monitored control of OSA on CPAP, enabling supervised withdrawal in carefully selected cases. While single-patient observations cannot establish causality, they complement emerging evidence and can guide patient counseling and hypothesis generation for prospective studies.

## 1. Introduction

The coexistence of T2DM and morbid obesity (BMI ≥40 kg/m²) presents multiple clinical challenges. Excess adiposity contributes to insulin resistance and complicates the management of hyperglycemia.^[1]^ Moreover, many individuals with severe obesity tend to develop obstructive sleep apnea (OSA), further increasing the cardiometabolic risk.^[2]^ In such patients, weight reduction has been shown to significantly improve both metabolic parameters and respiratory function.^[3]^ Conventional approaches to T2DM treatment, including lifestyle modifications and the use of oral antidiabetic agents and insulin, often have limited efficacy in the presence of severe obesity. Moreover, insulin therapy can promote weight gain and exacerbate metabolic dysfunctions. Recently, dual receptor agonists of GIP and GLP-1, such as tirzepatide, have emerged as promising therapeutic agents that offer substantial glycemic control and clinically significant weight loss.^[4]^

In 2024, randomized phase 3 trials (SURMOUNT-OSA) reported that tirzepatide reduced the apnea–hypopnea index (AHI) and body weight in adults with moderate-to-severe OSA and obesity,^[5]^ and on December 20, 2024, the U.S. Food and Drug Administration approved tirzepatide as the first medication indicated for OSA in adults with obesity.^[6]^ These developments provide a contemporary context for interpreting single-patient, real-world trajectories.

This case report describes a patient with T2DM (prior peak HbA1c 8.2%), class III obesity (BMI 40.7 kg/m²), and severe OSA who experienced significant weight loss, improved glycemic control, and improvement in sleep-disordered breathing following tirzepatide initiation. Because practical descriptions of pharmacologic weight-loss pathways that culminate in objective, device-monitored control enabling continuous positive airway pressure (CPAP) withdrawal remain limited, we outline the clinical decision-making that led to supervised discontinuation of CPAP. Tirzepatide was initiated in June 2023 for diabetes care with a weight-loss objective, before any OSA indication had been approved by the U.S. FDA, and OSA was reassessed using objective monitoring.

## 2. Case report

This case report included a 48-year-old man with T2DM, severe obesity, and OSA. The patient first presented to our cardiology department in February 2020 with complaints of headache and hypertension. At his initial visit, his height, weight, and body mass index were 178 cm, 129.0 kg, and 40.7 kg/m².

His medical history was notable for right testicular cancer diagnosed at the age of 20, for which he underwent orchiectomy. At age 41 years, he was diagnosed with hypertension and was initiated on antihypertensive therapy, although he discontinued treatment after 3 months. He had a 25-year smoking history of 20 cigarettes per day, but successfully stopped smoking at age 42. Furthermore, alcohol consumption was not reported. Appropriate antihypertensive therapy was initiated following the diagnosis of hypertension.

In January 2021, the patient was diagnosed with T2DM, had an HbA1c level of 7.0%, and was subsequently counseled on lifestyle modifications, emphasizing diet and exercise. The following month, he experienced marked daytime sleepiness. In March 2021, full-night in-laboratory polysomnography (PSG) using a PSG-1100 system (TEIJIN Ltd., Tokyo, Japan) during an inpatient admission at Hirose Hospital showed an AHI of 35 events/h (mean apnea duration 37 seconds; maximum 198 seconds), consistent with severe OSA. Baseline Epworth Sleepiness Scale score was 18. Thus, CPAP therapy was initiated in April 2021 and has been used every night since then. Auto-adjusting positive airway pressure therapy was delivered using a SleepMate 10 device (TEIJIN Ltd., Tokyo, Japan), initially set at 4 to 15 cm H_2_O with standard humidification. During therapy, device downloads showed an average leak of 2.4 L/min and a residual AHI of approximately 2 events/h, which remained stable over follow-up (2021–2023). Daytime sleepiness also improved, with the Epworth Sleepiness Scale decreasing to 3. Simultaneously, treatment with multiple oral hypoglycemic agents, including metformin and DPP-4 inhibitors, was initiated. Although the HbA1c level increased to 7.5% and his weight increased to 120 kg in August 2021, subsequent continuous nutritional counseling resulted in well-controlled glycemic levels and weight stabilization.

**Figure 1.**
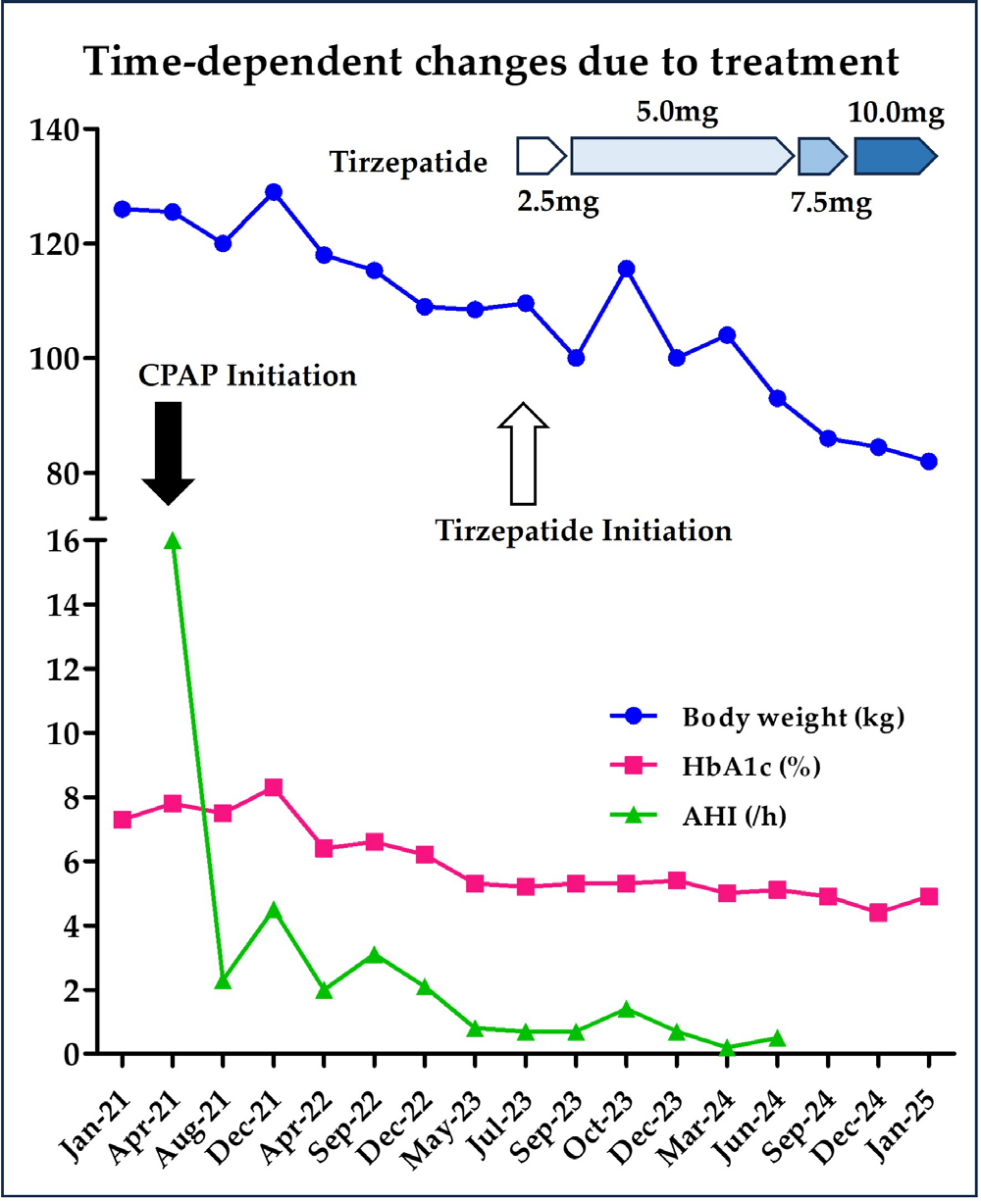
Time-dependent changes in body weight, HbA1c level, and residual AHI after continuous positive airway pressure and tirzepatide initiation. Post-initiation sleep metrics derive from CPAP telemonitoring (device-derived residual AHI under therapy; usage and leak tracked). Baseline diagnosis was established by in-lab PSG at Hirose Hospital. The black arrow indicates the initiation of CPAP therapy. The white arrow indicates tirzepatide initiation. Dose escalation of tirzepatide is also shown. AHI = apnea–hypopnea index, CPAP = continuous positive airway pressure, HbA1c = hemoglobin A1c, PSG = polysomnography.

CPAP therapy was maintained for 26 months after initiation (up to June 2023). However, subjective symptoms such as daytime fatigue and sleepiness persisted. Despite generally favorable glycemic control, the patient’s weight fluctuated between 108 and 129 kg without any significant reduction. Considering these issues, in June 2023, treatment with tirzepatide, a dual receptor agonist for GIP and GLP-1, was initiated at a low dose and gradually escalated to mitigate gastrointestinal adverse effects (nausea and diarrhea). Concurrently, moderate caloric restriction and mild-to-moderate physical activity were recommended. Under CPAP, the device-derived residual AHI remained low (~1 event/h). As the patient’s weight decreased, however, he began to report mask-related discomfort. We therefore hypothesized that his OSA might have remitted and arranged a reassessment. A home sleep apnea test (simplified polysomnography) in July 2024 showed a respiratory event index of 4.5 events/h, below the diagnostic threshold. Based on these findings and shared decision-making, CPAP was discontinued in August 2024. Longitudinal trajectories of body weight, HbA1c, and device-derived residual AHI while on CPAP are shown in Figure 1. The clinical course following the initiation of tirzepatide was favorable. Within 3 months, the patient experienced a 10 kg weight reduction, with his BMI improving from 40.7 to 34.1 kg/m². His HbA1c level stabilized at approximately 5%, indicating sustained glycemic control. However, continuous glucose monitoring was not obtained; HbA1c and fasting glucose were used to describe glycemic changes. Metabolic parameters included serial lipid measurements at 4 clinical time points, at type 2 diabetes diagnosis, after CPAP initiation, after tirzepatide initiation, and before CPAP cessation, as summarized in Table 1. Furthermore, the patient reported significant improvements in daytime sleepiness, fatigue, activity level, and concentration.

**Table 1:** Lipid profile across key time points.

Time point	Total cholesterol (mg/dL)	LDL-C (mg/dL)	HDL-C (mg/dL)	Triglycerides (mg/dL)
At DM diagnosis (January 2021)	205	113	39	523
Rosuvastatin 5 mg initiation (April 2023)	220	105	48	294
After tirzepatide initiation (June 2023)	145	66	47	223
Before CPAP cessation (August 2024)	104	53	39	126

Time points: at type 2 diabetes diagnosis; after CPAP initiation; after tirzepatide initiation; before CPAP cessation.

Parameters (mg/dL).

Rosuvastatin 5 mg once daily was initiated by another provider in April 2023 and continued without dose changes; lipid trends should be interpreted with caution as a potential confounder.

CPAP = continuous positive airway pressure, HDL-C = high-density lipoprotein cholesterol, LDL-C = low-density lipoprotein cholesterol, TC = total cholesterol, TG = triglycerides.

Adverse effects were limited to transient mild gastrointestinal symptoms during the initial phase of tirzepatide therapy, which resolved spontaneously without treatment discontinuation. No hypoglycemia or serious adverse events occurred.

Ultimately, his body weight decreased to 82 kg (BMI 25.9 kg/m²) and his HbA1c level has been maintained at ~4.9%, and he continues to have good glycemic control. Daily functioning remained stable, and no recurrence of daytime sleepiness was observed as of August 2025.

## 3. Discussion

This study reported a case showing significant improvement in both T2DM and OSA following diabetes treatment with tirzepatide, primarily aimed at weight reduction.^[7]^ This case emphasizes the potential effectiveness of tirzepatide in managing severe obesity complicated by OSA and T2DM.^[8]^ Tirzepatide simultaneously targets 2 incretin pathways (GIP and GLP-1), suggesting that it may provide favorable effects on weight loss and insulin sensitivity compared with conventional GLP-1 receptor agonists.^[9]^ Even among patients with inadequate glycemic control despite oral antidiabetic medications, tirzepatide significantly reduces body weight and glucose levels, potentially improving obesity-related complications.^[10]^ In our patient, CPAP telemonitoring documented persistently low device-derived residual AHI during therapy; combined with sustained weight loss and symptom improvement, this supported supervised discontinuation of CPAP. We recognize that device-derived values are not directly comparable to diagnostic AHI, so our interpretation remains descriptive. Furthermore, contemporary evidence has advanced rapidly: randomized phase 3 trials (SURMOUNT-OSA) showed that tirzepatide significantly reduced AHI and body weight in adults with moderate-to-severe OSA and obesity,^[5]^ and on December 20, 2024 the U.S. Food and Drug Administration approved tirzepatide as the first medication indicated for OSA in adults with obesity.^[6]^ Positioning our real-world observation against this backdrop provides implementation details complementary to trial efficacy (e.g., timing and conditions for CPAP withdrawal). Tirzepatide was started in June 2023 for T2DM and weight reduction, which predated the later U.S. FDA approval for OSA; our observation is therefore descriptive rather than inferential.

**Figure 2.**
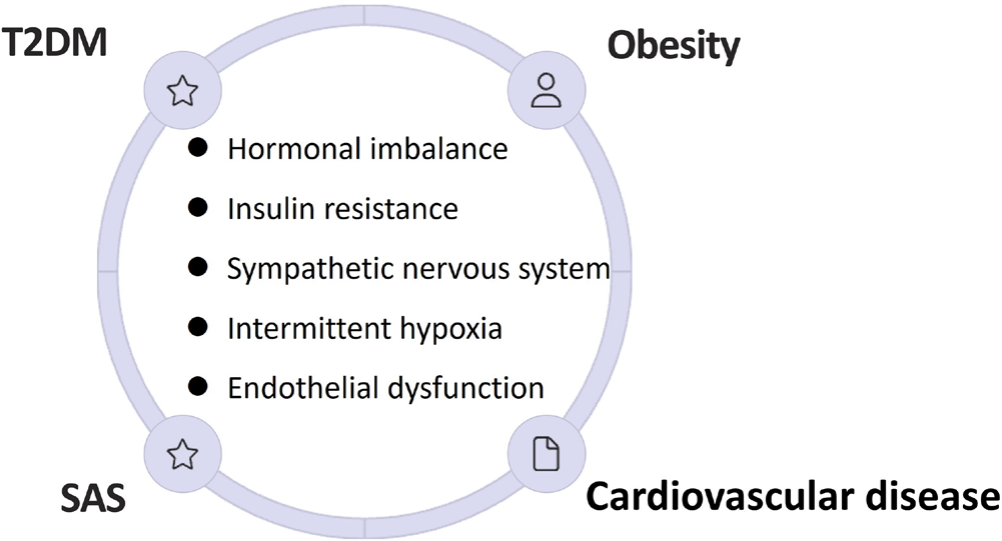
A schematic illustration of the bidirectional interactions between OSA and T2DM with obesity as a central mediator. OSA = obstructive sleep apnea, T2DM = type 2 diabetes mellitus.

The association between OSA and T2DM has been well established, and their co-occurrence is frequently observed clinically.^[11]^ Data from Western countries have indicated a prevalence of OSA in patients with T2DM ranging from 55% to 86%.^[12]^ Conversely, the prevalence of T2DM in OSA patients tends to be significantly higher than that in the general population, ranging from 26% to 30%.^[13]^ However, in Japan, the prevalence of OSA among T2DM patients remains relatively low at 7.2%.^[14]^ Another report also indicated that 44% of Japanese OSA patients were diagnosed with T2DM.^[15]^ Although obesity rates in Asia remain lower than those in Western countries, the prevalence is steadily rising. As shown in Figure 2, complex interactions exist between T2DM, OSA, obesity, and cardiovascular diseases.

Intermittent hypoxemia and fragmented sleep associated with OSA exacerbate insulin resistance, which negatively affects glycemic control. Activation of the sympathetic nervous system and heightened inflammatory responses due to OSA may increase the cardiovascular risk in diabetic patients.^[12]^

Weight reduction is widely recognized as a critical factor for improving OSA. Excess adipose tissue, particularly around the neck and upper airways, exacerbates airway collapse during sleep. A recent review reported that GLP-1 receptor agonists might significantly improve OSA symptoms not only by sustained weight loss, but also by reducing adiposity around the upper airway and suppressing systemic inflammation. Even modest weight loss can reduce apnea episodes and improve nocturnal oxygen saturation.^[16]^ In the present study, a substantial reduction in BMI correlated with significant improvements in OSA severity, which is consistent with the existing evidence. Mechanistically, reductions in peripharyngeal/tongue fat and systemic inflammation likely contributed, while any tirzepatide-specific effect beyond weight loss remains to be clarified.^[16,17]^

Despite the promising outcomes observed in this case, randomized controlled trials and long-term follow-up studies involving larger patient populations are required to determine the efficacy and safety of tirzepatide for managing patients with T2DM complicated by severe obesity and OSA. Importantly, decisions to withdraw CPAP should be individualized and based on objective testing (ideally in-lab off-CPAP polysomnography), with post-withdrawal monitoring for recurrence, particularly if weight is regained. Future research should address the potential adverse effects of tirzepatide treatment, patient adherence, and long-term cardiovascular outcomes. Clinicians should promote comprehensive management strategies that integrate pharmacotherapy with individualized dietary guidance, structured physical activity programs, weight management counseling, and consistent OSA treatment to optimize clinical outcomes and overall patient health.

## 4. Conclusion

This study revealed that tirzepatide therapy in a patient with morbid obesity, poorly controlled T2DM, and OSA resulted in significant weight loss, improved glycemic control, and enhanced sleep-disordered breathing. These outcomes suggest that tirzepatide administration may serve as a valuable therapeutic option for patients with obesity-related OSA and difficult-to-manage hyperglycemia. However, large-scale randomized controlled trials are needed to confirm these findings and assess the long-term sustainability, adherence, and safety of tirzepatide therapy in patients with T2DM and comorbid conditions, such as morbid obesity and OSA.

## Acknowledgments

The authors would like to thank the patient for granting permission to publish this case report and Enago (www.enago.jp) for English language review.

## Author contributions

**Supervision:** Naotake Funamizu, Tsunemichi Hirose.

**Writing – original draft:** Noriko Funamizu.

**Writing – review & editing:** Naotake Funamizu, Tsunemichi Hirose.
